# Gut microbiota and postoperative complications in colorectal surgery and its potential association with intestinal permeability and NLRP6 inflammasome

**DOI:** 10.3389/fimmu.2025.1701650

**Published:** 2026-01-26

**Authors:** Manuel Buitrago-Ruiz, Celia Arias-Sánchez, Maria Magdalena Asensio-López, Juan José Martínez-García, Victoriano Soria-Aledo, Graciela Valero-Navarro, Santiago Cuevas

**Affiliations:** 1 Department of General and Digestive Tract Surgery, Morales Meseguer General Hospital, Murcia, Spain; 2 BioMedical Research Institute of Murcia Pascual Parrilla (IMIB-FFIS), Murcia, Spain; 3 Department of Biochemestry and Molecular Biology B and Inmunology, Faculty of Medicine, University of Murcia, Murcia, Spain; 4 Department of Surgery, Pediatrics, Obstetrics and Gynecology. University of Murcia, Murcia, Spain

**Keywords:** anastomotic leak (AL), colorrectal surgery, inflammasome, inflammation, surgery, surgical complications, intestinal permeability, dysbiosis

## Abstract

This manuscript reviews the current evidence describing the relationships between surgical trauma, altered intestinal permeability, dysbiosis, inflammasome activation, and postoperative inflammation in the development of AL. Surgical tissue disruption and anastomosis creation can modify the intestinal microbiota and compromise epithelial barrier function, promoting a proinflammatory environment mediated by inflammasomes such as NLRP3, NLRC4, and NLRP6. This review analyses the inflammatory pathways that may regulate intestinal homeostasis, their potential contribution to postoperative complications such as AL, and how these insights may guide future preventive strategies or help identify patients at increased risk.

## 1 Introduction, the pathology of anastomotic leakage in colorectal surgery is a complex and multifaceted phenomenon

Anastomotic leak (AL) is a potentially serious complication that can occur after intestinal surgery, and it is one of the most clinically significant complications in colorectal surgery due to its severity and frequency (1). Despite advances in surgical technique, perioperative care and patient optimisation, reported AL rates vary widely across the literature. Overall, colorectal resection related AL incidence ranges approximately 2% to 19% (2, 3). When stratified by anatomical level distal rectal and colo-anal anastomoses present higher rates (up to 18–19%), whereas right-sided colectomies where rates are as low as 1–3% (2, 3).

In efforts to minimise the occurrence of AL following colorectal surgery, multiple preventive strategies have been adopted spanning the pre-operative, intra-operative and post-operative periods. Nevertheless none of these interventions guarantees that AL will be entirely prevented. AL remains multifactorial, and even when best practices are applied a residual risk persists (4–6). In recent years, AL incidence, along other surgical complications, appears to have reached a plateau, suggesting the existence of relevant yet incompletely understood contributing factors to AL that are only now beginning to be recognised (7).

Our previous studies have analyzed the value of inflammation as a predictor of AL before clinical onset (3, 8), supporting the utility of inflammatory biomarkers for early diagnosis. The clinical onset of AL is characterized by abdominal pain, which can be either localized or generalized when diagnosed late and often accompanied by signs of acute peritonitis, fever and elevated leukocyte counts. Other symptoms, such as ileus, obstruction, or sepsis, are less common but may indicate a more severe leak. Importantly, AL may be present for 24–48 hours before symptoms arise, with most cases becoming clinically apparent around the fifth or sixth postoperative day (9).

Our group has demonstrated that an elevation in C Reactive Protein (CRP) above 15 mg/dL on the third postoperative day (10, 11) is an important predictor of AL, representing a valuable tool for early detection and prevention of its associated morbidity. However, the biological mechanisms that underlie AL and other postoperative complications are not yet well defined. Previous authors have suggested that, when technical errors are controlled, AL results from a complex and dynamic interaction of several factors and biological processes, in which inflammation and the immune system response play a significant role (11, 12). In this context, understanding the molecular pathways triggering inflammation and inflammasome activation may therefore be essential for developing new preventive or therapeutic strategies.

As previously mentioned, a sustained elevation of CRP between postoperative days three and five serves as an early warning sign of complications, including AL NLRP3 inflammasome activation is known to contribute to CRP upregulation (13, 14), however, the precise inflammatory pathways driving CRP elevation in AL, and whether damage associated molecular patterns (DAMP) release plays a direct role, remain unclear. While inflammation is already being used as a clinical marker for surgical complications, emerging evidence suggests that inflammatory modulators could serve as potential therapeutic tools to prevent AL (15, 16).

The activation of inflammasomes and persistent inflammation has been linked to tissue fibrosis and organ dysfunction, notably in the liver and kidneys. In hepatic tissue, cellular damage triggers the release of inflammatory mediators, prompting immune cell infiltration. Resident Kupffer cells are activated, leading to the secretion of proinflammatory cytokines such as IL-1 and IL-18. These cytokines, in turn, stimulate hepatic stellate cells, which differentiate into myofibroblasts and produce excessive extracellular matrix, contributing to fibrosis (17, 18). Similar mechanisms may be triggered after major surgical procedures. Murine models have shown that extensive surgical trauma leads to significant extracellular ATP release, a well-established DAMP (19). ATP further activates the inflammasome cascade via the P2X7 receptor and K+ ionophore, serving as a secondary trigger for NLRP3 inflammasome activation.

Macrophages activated through inflammasome pathways sustain inflammation and promote fibrotic tissue deposition. The STING pathway-mediated activation of macrophage XBP1 drives inflammasome activity and increases the production of TNF-α and TGF-β. When these cytokines interact with hepatocytes, fibroblasts, myofibroblasts, or stellate cells, they enhance extracellular matrix secretion, potentially leading to fibrosis (15, 20). In uncontrolled cases, fibrosis can result in postoperative complications, such as excessive tissue formation following spinal surgery, causing radiculopathy or lumbar canal stenosis (21). Similarly, in strabismus surgery, excessive fibrosis may lead to suboptimal surgical outcomes, making it a key therapeutic target (22). These findings suggest a possible connection between postoperative inflammatory dysregulation, fibrosis-related impairment of anastomotic healing, and AL (13, 15, 18, 23, 24).

This manuscript reviews the current evidence describing the relationships between surgical trauma, altered intestinal permeability, dysbiosis, inflammasome activation, and postoperative inflammation in the development of AL. Surgical tissue disruption and anastomosis creation can modify the intestinal microbiota and compromise epithelial barrier function, promoting a proinflammatory environment mediated by inflammasomes such as NLRP3, NLRC4, and NLRP6. This review analyses the inflammatory pathways that may regulate intestinal homeostasis, their potential contribution to postoperative complications such as AL, and how these insights may guide future preventive strategies or help identify patients at increased risk.

## 2 Inflammasome in the inflammatory response

The main way in which the innate immune system responds to infection and tissue injury is through the development ofan effective inflammatory response. This response is mediated by large protein complexes expressed on immune cells. These are specific intracellular molecular structures that induce caspase-1 activation, which triggers the processing and activation of pro-inflammatory cytokines such as IL-1β and IL-18 (25).

Over the last decade, Polly Matzinger’s “Danger Hypothesis” (26) has gained prominence, providing a new conceptual framework for the activation of the innate and adaptive immune systems. The innate system is primed to sense “danger signals, “ described as DAMPs, and to respond to them by activating inflammation and shaping adaptive immunity. DAMPs are endogenous molecules that, when released into extracellular or otherwise inappropriate compartments, have potent pro-inflammatory effects and signal tissue damage, such as that occurring during surgical procedures (13). These danger signals, may be released due to cellular and tissue rupture after surgery, inducing the activation of the NLRP3 inflammasome, leading to uncontrolled tissue fibrosis, and potentially contributeto the progression of AL and other observed postoperative complications (13, 27).

Inflammation begins with the recognition of pathogen-associated molecular patterns (PAMPs) DAMPs by pattern recognition receptors (PRRs), extracellular toll-like recptors (TLRs) and intracellular, NOD-like receptors (NLRs) (28). Many NLRs assemble and form multi-protein complexes that regulate inflammation at the post-translational level;these complexes are known as inflammasomes (28). In general, inflammasomes are composed of an NLR (sensor protein), an ASC adaptor (adaptor protein containing a caspase activation and recruitment domain, known as CARD) and a cysteine protease caspase-1 (effector protein) (28).

The NLR sensor comprises three main domains, an N-terminal effector domain, a central nucleotide-binding domain and a C-terminal leucine-rich repeat domain (29). Importantly, NLRs are divided into two subgroups based on differences in the N-terminal effector domain. NLRPs, which contain a pyrin domain (PYD), and NLRC, which contain a caspase activation and recruitment domain (CARD) (29). NLR receptors that mediate inflammasome assembly include NLRP-1, 3, 6, 7, 9 and NLRC-4 (30).

The interaction between a sensor protein and its specific activator leads to inflammasome assembly (31). Activated inflammasomes induce the maturation of pro-inflammatory cytokines that recruit immune cells to the site of infection and injury, triggering inflammation and promoting tissue and organ repair (31, 32). More specifically, following recognition of PAMPs orDAMPs by NLR drives inflammasome oligomerisation, allowing recruitment of the ASC adaptor, which in turn recruits procaspase-1 via CARD–CARD interactions (33). This facilitates autoproteolysis of procaspase-1 into activated caspase-1, which cleaves pro-IL-1β and pro-IL-18 into their mature forms. Mature IL-1β binds to its receptor and their interaction triggers lymphocyte activation, creating a pro-inflammatory environment. Mature IL-18 induces the production of cellular stress-related components, further promoting the chemotactic environment (secretion of inflammatory factors and chemokines) and recruitment of immune cells (32).

In addition, caspase-1 induces the cleavage of gasdermin D (GSDMD) in its activated form, which forms transmembrane pores that facilitate the release of intracellular contents, thereby triggering a type of inflammation-related cell death known as pyroptosis (33). Inflammasome-induced pyroptosis aims to destroy and eliminate infected or damaged cells in order to restore or maintain tissue homeostasis once the inflammatory response is over (31).

### 2.1 NLRP6 inflammasome in the intestine

The NLRP6 inflammasome is highly present in the gut immune system, and its functions are not yet fully elucidated. NLRP6 activation promotes the maturation of IL-1β and IL-18, thereby contributing to a pro-inflammatory environment (34–36). Additionally, NLRP6 regulates microbiota through the production of goblet cell mucus (37, 38), and it’s essential for maintenance of intestinal homeostasis (39). Moreover, NLRP6 has been implicated in the regulation of a healthy microbiome, intestinal barrier integrity (37, 40–43), and the pathogenesis of colorectal cancer and inflammatory bowel disease (34, 35, 44);

Direct activators of the NLRP6 inflammasome include short-chain fatty acids (SCFAs), gastrointestinal hormones, and precursors of bioactive molecules, along with intestinal dysbiosis (34).

The NLRP6 inflammasome is tightly linked to inflammation and dysbiosis, having several functions in the gut (35, 36, 38, 42, 45–48). Although NLRP6-dependent pathways are not fully defined, several authors propose that NLRP6 helps control pathogenic bacteria via multiple downstream effectors. NLRP6 activation increase mucus secretion, leading to microbiota control (37, 38, 42, 49). Moreover, NLRP6 activation may lead to altered intestinal permeability, which allow bacterial and intraluminal molecules, that may act as DAMPs or PAMPs, igniting the inflammatory cascade (7, 37, 38, 41, 42, 47, 49, 50). This gut inflammation then, spreads systemically, increasing the production of proinflammatory cytokines, such as IL-1 and IL-18, which lead to uncontrolled fibrosis, incorrect healing and AL (13, 15, 18, 21–24). NLRP6 also activates pathways that lead to an increase in IL-1 and IL-18, so it could be a direct activator of this inflammatory cascade, potentially lead to AL (35, 36, 38, 45, 48).

Intestinal diseases, such as colorectal cancer and inflammatory bowel disease (IBD), has been also directly linked to up- or downregulations of the expression of NLRP6 inflammasome (35, 44, 45, 51). NLRP6-deficient mice showreduced IL-18 production which led to alterations in gut microbiota (GM), with an increase in Bacteriodes strains. These mice developed mucosal hyperplasia, accelerated inflammatory cell recruitment, facilitated by an alteration in permeability, and exacerbation of colitis induced by dextran sodium sulfate (45). NLRP6 has also been associated with detrimental effects on epithelial cells in the gut. These different actions may respond to post-translational modifications such as ubiquitination or phosphorylation (35, 38, 48).

NLRP6 components are upregulated in response to microbial colonisation during early life, suggesting a key role for the microbiota in the induction of NLRP6-dependent antimicrobial responses and in shaping host–microbiota interaction (52). When dysbiosis is present, NLRP6-mediated intestinal homeostasis is downregulatedAs a result, the host becomes functionally deficient in IL-18 and downstream antimicrobial peptides, which facilitates the persistence of an aberrant microbiome (52). Moreover, dysbiosis and increased proximal epithelial colonization in NLRP6-deficient mice triggers enhanced translocation of microbial products, contributing to increased susceptibility to the development of colitis and colitis-associated colorectal cancer (52). NLRP6 is therefore critical in preventing aberrant microbiota-host interactions, which in turn are necessary to prevent adverse metabolic consequences.

This evidence shows a strong link between inflammation and NLRP6 activity in the physiological response to injury, this being either surgical or infectious. NLRP6 activation, combined with activation in other inflammasomes, such as NLRP3 and NLRC4, which are also present in the gut (7, 13, 35, 36, 41, 45, 48, 53), may play a role in AL and are reciprocal since its response may be triggered by surgical trauma.

### 2.2 NLRC4 inflammasome in the intestine

The NLRC4 inflammasome is yet another inflammasome that regulates GM and may have a potential role in dysbiosis phenomena and inflammation (49, 53). The NLRC4 inflammasome is primarily related to gram-negative bacteria (49, 53, 54). In a study by *Sofia Nordlander and colleagues*, NLRC4 expression in intestinal epithelial cells was found to be important for protection against intestinal pathogens (55). A gain-of-function mutation has been described as a cause of autoinflammatory disease associated with dysbiosis (54). To characterise the role of NLRC4 in bacterial inflammation, Nordlander et all used the mouse pathogen *Citrobacter rodentium*. This is a gram-negative bacterium similar to human enterohaemorrhagic pathogens (*E. coli*) (56). The pathology associated with this bacterium mimics certain features seen in patients with inflammatory bowel disease (IBD). Interestingly, these patients have been found to have increased levels of bacteria such as *E. coli* in the terminal ileum (56), supporting a link between epithelial infection and intestinal inflammation.

To investigate the role of the NLRC4 inflammasome, they used wild-type (WT) and NLRC4 knock-out (NLRC4 -/-) B6 mice, all infected with *C. rodentium*. Their results showed that knockout mice exhibited, an increased systemic immune response; a marked exacerbation of the pathological features of intestinal inflammation, such as hyperplasia, leukocyte infiltration and oedema; hyperproliferation of epithelial cells in the colon; and increased levels of colonisation, as these animals had significantly increased loads of tissue-adherent *C. rodentium* in the cecum. This was compared to WT mice. All these results demonstrate that NLRC4-mediated protection limits early bacterial colonisation and subsequent intestinal inflammation, suggesting that NLRC4 activation is a critical component of early innate defense against intestinal bacterial pathogens (55).

### 2.3 NLRP3 inflammasomme

In a similar experimental model, Song-Zhao et al. demonstrated that NLRP3 plays a role analogous to NLRC4 in host responses to intestinal bacterial pathogens. To investigate the role of the NLRP3 inflammasome in bacterial-mediated intestinal inflammation, they infected cohorts of WT, NLRP3 knockout and ASC knockout mice with *Citrobacter rodentium*. They found that the absence of NLRP3 and ASC expression in the mice resulted in, severe colitis characterized by submucosal inflammation and leukocyte infiltration; systemic colonization and translocation; and increased intestinal inflammation and weight loss compared to WT. These findings show that NLRP3, like NLRC4, estricts intestinal inflammation, limits bacterial localisation in the gut, and prevents severe pathology (57).

In this context, inflammasomes orchestrate immune tolerance or the induction of inflammatory responses to changes in the GM, highlighting the important role that each plays in regulating gut homeostasis and balance within the microbiome (58).

## 3 Microbiota, dysbiosis, relationship with inflammation and intestinal permeability

Microbiota is defined as the diverse group of living microorganisms, including bacteria, fungi, viruses, and archaea, that naturally colonize body surfaces such as the gastrointestinal tract, skin, respiratory pathways, and other mucosae. These microorganisms play essential roles in the host’s homeostasis and health, actively contributing to key processes such as nutrient metabolism, digestion, immune system modulation, and pathogen protection through the production of antimicrobial metabolites (59). The intestinal tract is colonized by many bacteria that are continuously exposed to a wide variety of antigens and microbial products. This requires a tightly regulated balance between mucosal immune tolerance toward commensals and robust responses against pathogens (58).

The physiological GM is yet to be completely standardized, but there is evidence that plays a central role in both several human physiological pathways and pathologies (46, 60). Surgery, both as a direct anatomical insult and through associated perioperative management (antibiotics, fasting, bowel preparation, anaesthesia), can profoundly affect GM homeostasis (7, 47, 50, 61, 62). This relation is reciprocal since dysbiosis can have a direct impact in AL. It has been described that mice infected with an Enterococcus faecalis strain that produces a collagenase called matrix metalloprotease 9 (MMP9) are more likely to develop AL, and when treated with inhibitors or the E. faecalis strain is eliminated, AL incidence is reduced (7, 63).

### 3.1 Gut microbiota and dysbiosis

The human gastrointestinal tract hosts a wide and complex microbial community, which has been studied as one of the most important for maintaining human health. The GM is primarily composed of six bacterial phyla, *Firmicutes, Bacteroidetes, Actinobacteria, Proteobacteria, Fusobacteria*, and *Verrucomicrobia*, with the first two being the predominant groups (64). Under healthy conditions, the microbiota exhibits stable composition, resilience to disturbances, and an effective symbiotic interaction with the host. A healthy microbiota usually shows high taxonomic diversity, extensive microbial gene richness, and a stable core microbiota (65).

The GM performs multiple essential functions in the host’s physiology. It plays a crucial role in nutrient extraction and biosynthesis of bioactive molecules, including vitamins, amino acids, and essential lipids. Additionally, it ensures proper immune function, not only protecting the host from external pathogens by producing antimicrobial substances but also actively participating in the development and maturation of the intestinal epithelium and immune system, regulating the mucus layer, lymphoid structures, and lymphocyte activity (66, 67).

The balance between the host and the GM is essential for maintaining human health. However, when there is a significant imbalance in the microbiota’s composition, where beneficial bacteria are replaced by potentially pathogenic microorganisms, the gut becomes more susceptible to infections and damage. This phenomenon, known as dysbiosis, refers to the alteration of intestinal homeostasis, marked by decreased microbial diversity and increased proliferation of pathogenic bacteria (68). GM composition is shaped by diet, medication use, host immunity, epithelial status, and by the microbiota itself. Because of the resilience of the GM, a single factor is often insufficient to cause dysbiosis, whereas the combined action of multiple factors, such as inflammation, genetic susceptibility, lifestyle, or drugs that can significantly alter microbial communities and promote imbalance (69).

### 3.2 Dysbiosis, inflammation, and intestinal permeability

Intestinal homeostasis is regulated by the interaction between the intestinal epithelium, the microbiome, and the host’s immune system. This system heavily depends on the integrity of the epithelium, reinforced by binding proteins like tight junctions (TJs), desmosomes, and adherens junctions (70). A balanced microbiome plays a crucial role in maintaining the intestinal barrier, which protects the host from luminal antigens, pathogens, and toxins while allowing selective permeability (71).

Microbial components and their metabolites are primarily recognized by pattern recognition receptors (PRRs), such as Toll-like receptors (TLRs) and NOD-like receptors (NLRs). Signaling pathway through TLR2 is essential to preserve the integrity of TJs under normal conditions. However, improper regulation of this pathway can activate inflammation via the nuclear factor-kappa B (NF-κB) pathway, triggering the production of pro-inflammatory cytokines such as interferon gamma (IFN-γ) and tumor necrosis factor-alpha (TNF-α). These cytokines alter TJs, increasing intestinal permeability and creating a pro-inflammatory environment (72).

In contrast, short-chain fatty acids (SCFAs), such as acetate, propionate, and butyrate, are products of fiber fermentation by beneficial gut bacteria like Bacteroides. These SCFAs are essential for barrier function, as they promote balanced immune responses, support microbial homeostasis, and aid in the regeneration of mucosal tissue (73).

Dysbiosis has been linked to various gastrointestinal diseases and disorders, including inflammatory bowel disease (IBD) and irritable bowel syndrome (73). In patients with IBD, a significant reduction in beneficial bacteria like *Faecalibacterium prausnitzii* has been observed, along with an increase in pro-inflammatory bacteria such as *Proteobacteria* and adherent-invasive *Escherichia coli*. This alteration reduces the production of butyrate, an essential metabolite for epithelial health, and promotes an exaggerated immune response that damages the intestinal mucosa, increasing permeability (70, 74).

In dysbiosis conditions, an increase in lipopolysaccharide (LPS)-producing Gram-negative bacteria is also observed, such as *Proteobacteria*, worsens the situation. LPS is a potent activator of TLR4, which activates the NF-κB pathway, leading to the release of pro-inflammatory cytokines, perpetuating the inflammation process (70). In experimental models, such as germ-free (GF) mice, the absence of microbiota significantly alters the intestinal immune system. These animals show a reduction in IgA-producing plasma cells and CD4+ T cells, key elements for mucosal immune defense. Additionally, the lack of microbiota affects the normal development of intestinal epithelial cells, compromising the integrity of the intestinal barrier. As a result, GF mice are more susceptible to infections by bacteria, viruses, and parasites (72, 75).

In summary, dysbiosis, inflammation, and increased intestinal permeability can create a harmful self-perpetuating cycle. First, dysbiosis activates the intestinal immune system, triggering an inflammatory response. This inflammation, in turn, affects the integrity of TJs between epithelial cells, leading to increased intestinal permeability. With the intestinal barrier compromised, toxins and microorganisms can cross into the bloodstream, intensifying dysbiosis and exacerbating inflammation. Maintaining a balanced GMis essential not only for intestinal health but also to prevent systemic inflammatory conditions arising from a “leaky gut”.

### 3.3 Microbiota and surgery

In any surgical intervention affecting the gastrointestinal tract, the function of this barrier is compromised, potentially affecting its ability to preserve intestinal homeostasis.

Surgical interventions themselves profoundly alter the GM, creating a pro-inflammatory microenvironment that further impairs anastomotic healing. Skowron et al. reported significant shifts in microbial composition following surgery, including an increase in pathogens like Escherichia coli and Enterococcus, which adhere to the anastomotic site and exacerbate local inflammation (76). Several authors emphasise that these microbial changes, together with surgical stress and ischaemia, significantly contribute to AL and long-term functional impairment (11, 77–80).This dysbiotic state has been linked to inflammasome activation, specifically through NRLC4 and NLRP6, both of which are associated with increased intestinal permeability and impaired barrier function (40, 47–50, 53, 54, 61, 81).

Reduced microbial diversity and overrepresentation of pathogenic bacteria have been strongly associated with poor anastomotic healing (5, 7, 77–80). There are multiple reviews that describe how bacteria such as Enterococcus faecalis and Pseudomonas aeruginosa produce collagenases that degrade the extracellular matrix and activate matrix metalloproteinase-9 (MMP9), disrupting tissue integrity and increasing the risk of AL (7, 11, 82, 83). Also, higher levels of mucin-degrading bacteria like Lachnospiraceae and Bacteroidaceae, significantly correlates with increased AL risk (84, 85). In contrast, SCFAs—particularly butyrate—show protective effects on anastomotic integrity (86, 87). Studies also emphasize the importance of preoperative preparation, combining oral antibiotics and mechanical bowel preparation, which reduces AL rates compared to either method alone (5, 7). Foppa et al, highlight the need for microbiota-targeted therapies to mitigate this complication by addressing the underlying biological mechanisms (11).

Interventions aimed at restoring microbial balance have shown promise in reducing postoperative complications. Darbandi et al. found evidence which suggest that probiotics enhance gut barrier integrity, promote microbial diversity, and decrease the prevalence of pathogenic strains, all of which may lower the incidence of AL (88, 89)​. Evidence suggests that preoperative and perioperative microbiota-targeted strategies, such as probiotics, could enhance mucosal healing, immune modulation, and reduce pathogenic colonization.

Liu et al, concluded that patients treated with probiotics showed less infectious complications after colorectal surgery. Patients that were not treated with probiotics had decreased colonization by Paseudomona, Candida and Enterobacteriae phyla (90). Darbandi et al. found similar results when reviewing clinical trials that showed the effects of probiotics in colorectal surgery patients (91). Polakowski et al. found that synbiotics could decrease postsurgical infectious complications, and reduced both IL-6 and CRP levels compared to placebo (92).

Collectively, these data demonstrate a strong link between surgery, dysbiosis, and postoperative complications, including AL, even in patients without classical risk factors. Moreover, therapies targeting this axis have shown encouraging results in reducing AL incidence and other adverse outcomes. Our aim is to explore the molecular pathways that underlie these observations and to integrate them with the pro-inflammatory state driven by NLRP6 and NLRC4 inflammasome activation after intestinal surgical injury.

## 4 Proposed mechanisms of pathophysiology on intestinal leakage, dysbiosis and fibrosis; the role of inflammation

Intestinal anastomosis is a common procedure in most patients undergoing colorectal surgery that involves bowel resection. One of the most serious complications arising in the early postoperative period is anastomotic leakage (AL), which can necessitate admission to the intensive care unit, require reoperation, or even result in mortality. Given its impact, identifying the factors that influence AL and developing tools for early detection and prevention remain key priorities in colorectal surgery (8).

Surgery necessarily involves breaking the intestinal barrier, colonized by various populations that make up the microbiota (78). Scientific evidence suggests a relationship between AL and dysbiosis. Although the mechanisms are not fully understood, various perioperative procedures have been associated with altering the phenotypes and genotypes of commensal bacteria, promoting their transformation into invasive pathogens that degrade tissues during surgery, leading to more overall complications (93). In this context, the reduction of SCFAs, along with an increase in pro-inflammatory substances such as LPS, has been closely linked to the occurrence of anastomotic leakage (94). Factors such as antibiotic use, perisurgical intestinal preparation, dietary changes, or pre-existing inflammatory conditions can cause dysbiosis, reducing the gut’s ability to heal properly and promoting the occurrence of postoperative complications, such as anastomotic leakage. Therefore, GM before and after surgery may be crucial for predicting or reducing the risk of this complication.

**Figure 1:**
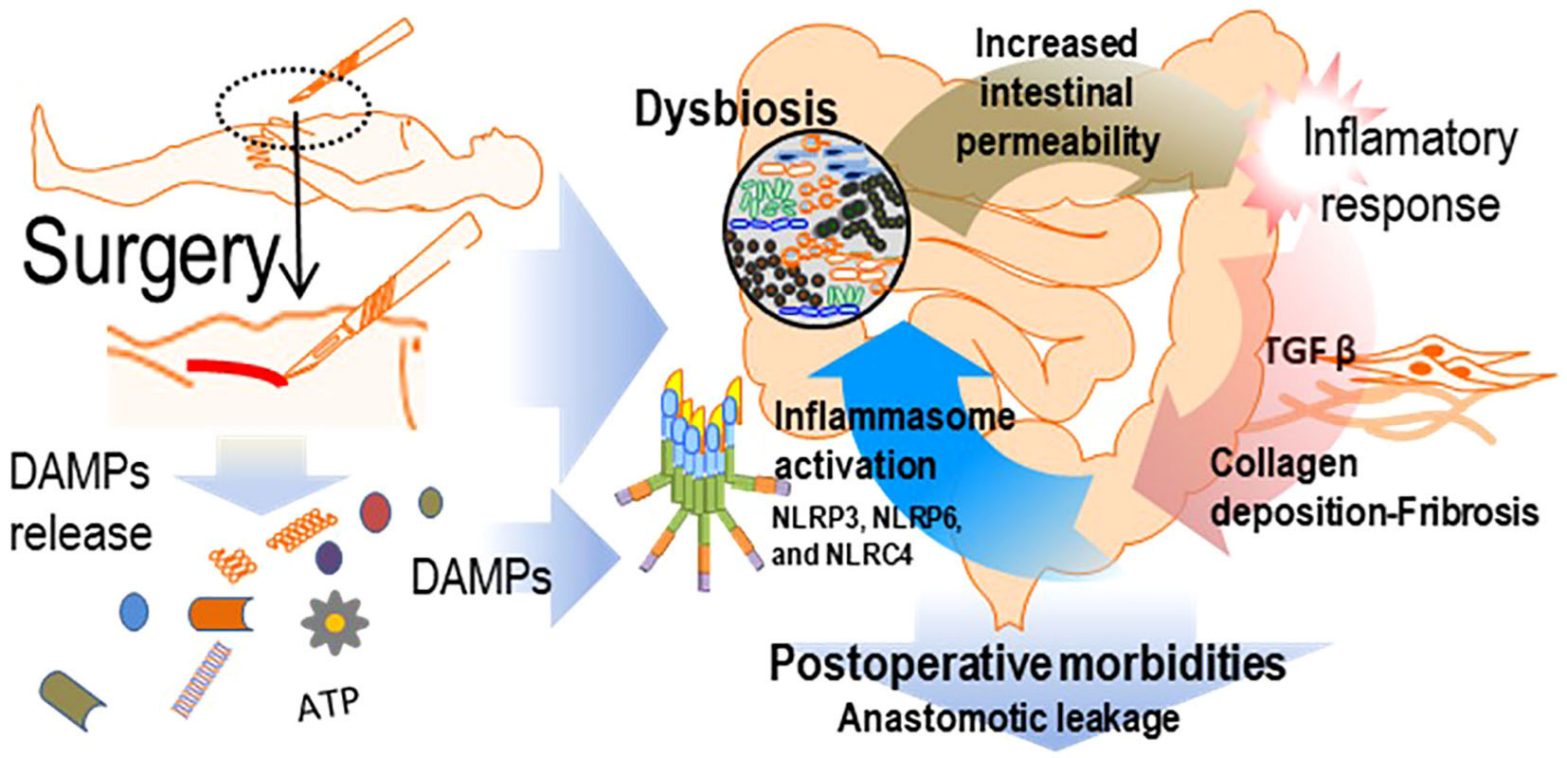
Inflammation, fibrosis and intestinal permeability may be associated with postoperative morbidities in intestinal surgery. Damage-Associated Molecular Patterns (DAMPs) are released into the extracellular space during surgery and could activate inflammasomes, triggering inflammation that may affect the gut microbiota. Dysbiosis has been associated with increased intestinal permeability, systemic inflammation and intestinal fibrosis, creating a feedback loop of inflammatory activation that could lead to anastomotic leakage and other post-operative complications.

This manuscript also describes several pathways through which inflammation may be activated in the postoperative setting (**Figure 1**). Inflammation is fundamental for maintaining immune balance in intestinal tissues and preserving barrier integrity (72). Growing evidence suggests that multiple inflammasomes may be involved in triggering inflammatory processes within this tissue, thereby promoting fibrosis. This, in turn, could impair postoperative tissue sealing and lead to anastomotic leakage.

Identifying universal mechanism underlying all cases of AL is unlikely. However, current evidence highlights a synergistic interaction between inflammation, the intestinal microbiota, and, in particular, the activity of the NLRP6 and NLRP3 inflammasomes. Alterations in inflammasome activation or changes in the microbiota can simultaneously affect both systems, generating a pathological environment that promotes fibrosis and leakage.

Therefore, a detailed study of these factors, their interrelationships, and how the immune system influences GM (and vice versa) is essential to explore his effect triggering AL. Both dysbiosis and gut inflammation may disrupt intestinal barrier integrity, allowing PAMPs to translocate into tissues and promote systemic inflammation through the activation of NLRP3 and NLRP6 inflammasomes. This inflammatory response can, in turn, alter the GM composition, creating a self-perpetuating cycle of dysbiosis and inflammation. These mechanisms warrant precise investigation, as the evidence suggests they could play an important role in the pathogenesis of post-surgical complications, or even predict the occurrence of anastomotic leakage in some of the patients included in this study.

In this regard, the analysis of DAMP and PAMP concentrations in peripheral blood may serve as a promising biomarker and potential diagnostic tool. If confirmed, these circulating molecules released through inflammasome activity (for example, in renal tissue) could play a crucial role in the early identification of AL, allowing for timely preventive intervention to prevent this complication.

Additionally, inflammation is essential for tissue repair, uncontrolled activation or inhibition can lead to fibrosis and tissue damage. Given the potential role of inflammasome activation, via either DAMP release or dysbiosis, in AL following colorectal surgery, targeting these pathways may represent a promising avenue for both early diagnosis and therapeutic intervention.

## 5 Conclusions and future perspectives

Emerging evidence highlights that maintaining a healthy microbiota and a properly regulated inflammatory environment, including the proper activity of the gut NLRP6 inflammasome, may essential for preventing these conditions and for mitigating complications such as anastomotic leakage following gastrointestinal surgery. Managing the microbiota before and after surgical interventions may significantly improve recovery outcomes and reduce associated risks.

Future research should focus on elucidating the molecular mechanisms underlying the relationship between microbiota, dysbiosis, and systemic inflammation to develop targeted therapies for gastrointestinal disorders and related systemic diseases. Innovative approaches, such as microbiota modulation using prebiotics, probiotics, postbiotics, and fecal microbiota transplantation (FMT), as well as NLRP6 inflammasome regulation, hold significant promise for restoring gut balance and enhancing intestinal barrier function. Additionally, a deeper understanding of the GM’s role in postoperative recovery, its interaction with inflammatory cells, and inflammasome activity, particularly in preventing complications like anastomotic leakage, could revolutionize perioperative care in gastrointestinal surgery.

Finally, integrating this new knowledge about the role of inflammasome activity in the management of GM, its interactions and consequences on gut permeability, and its role in gut complications paves the way for precision medicine strategies to effectively manage dysbiosis-associated diseases and prevent postsurgery complications.

## Data Availability

The original contributions presented in the study are included in the article/supplementary material. Further inquiries can be directed to the corresponding authors.

## Abbreviations

AL: Anastomotic Leakage
DAMPs: Damage-Associated Molecular Patterns
CRP: C Reactive Protein
DNA: Deoxyribonucleic acid
IL-1β: Interleukin 1 beta
IL-18: Interleukin 18
NLRs: Nucleotide-binding domain and leucine-rich repeat receptors
ALRs: Absent in melanoma 2-like receptors
ASC: Apoptosis-associated speck-like protein
CARD: Caspase Activation and Recruitment Domain
GSDMD: Gasdermin D
HSCs: Hepatic Stellate Cells
TLR: Toll-like receptor
PAMPs: Pathogen-associated molecular patterns
HAMPs: Homeostasis-altering molecular processes
NF-κB: Nuclear factor kappa B
PRRs: Pattern Recognition Receptors
CLR: C-type Lectin Receptor
RLR: RIG-I-like receptor
NLR: NOD-like receptor
TAK1: Transforming growth factor β-activated kinase 1
TAB1: TGF-Beta Activated Kinase 1 (MAP3K7) Binding Protein 1
TAB2: TGF-Beta Activated Kinase 1 (MAP3K7) Binding Protein 2
IKK: IkappaB Kinase
IL-1: Interleukin 1
IL-6: Interleukin 6
IL-12: Interleukin 12
TNF-α: Tumor necrosis factor alfa
NACHT: Nucleotide-binding oligomerization core domain
LRR domain: Leucine-rich repeat
STAT: Signal Transducer and Activator of Transcription
RAGE: Receptor for Advanced Glycation End-products
TLR4: Toll-like receptor 4
TLR2: Toll-like receptor 2
TLR9: Toll-like receptor 9
P2X4R: The purinergic P2X4 receptor
P2X7R: The purinergic P2X7 receptor
mtDNA: Mitochondrial DNA
ROS: Reactive Oxygen Species
AIM2: Absent in melanoma 2
LDL oxidase: Low-density lipoprotein oxidase
FASN: Fatty acid synthase enzyme
RvD2: Resolvin D2
XBP1: X-box binding protein 1
STING: Stimulator of interferon gene
TGF-β: Transforming growth factor beta
µl: Microliter
HIPEC: Hyperthermic intraperitoneal chemotherapy
MOF: Multiple Organ Failure
SOFA: Sequential Organ Failure Assessment
